# Intermedin facilitates hepatocellular carcinoma cell survival and invasion via ERK1/2-EGR1/DDIT3 signaling cascade

**DOI:** 10.1038/s41598-020-80066-x

**Published:** 2021-01-12

**Authors:** Fei Xiao, Hongyu Li, Zhongxue Feng, Luping Huang, Lingmiao Kong, Min Li, Denian Wang, Fei Liu, Zhijun Zhu, Yong’gang Wei, Wei Zhang

**Affiliations:** 1 Department of Intensive Care Unit of Gynecology and Obstetrics, West China Second University Hospital, Sichuan University, Chengdu, People’s Republic of China; 2 Liver Transplantation Center, Beijing Friendship Hospital, Capital Medical University, Beijing, People’s Republic of China; 3 Department of Critical Care Medicine, State Key Laboratory of Biotherapy and Cancer Center, West China Hospital, Sichuan University and Collaborative Innovation Center of Biotherapy, No. 1, Ke Yuan 4th Road, Gao Peng Street, Chengdu, 610041 Sichuan People’s Republic of China; 4 Department of Liver Surgery, West China Hospital, Sichuan University, No. 1, Ke Yuan 4th Road, Gao Peng Street, Chengdu, 610041 Sichuan People’s Republic of China

**Keywords:** Cancer, Cell biology

## Abstract

As one of the most malignant cancer types, hepatocellular carcinoma (HCC) is highly invasive and capable of metastasizing to distant organs. Intermedin (IMD), an endogenous peptide belonging to the calcitonin family, has been suggested playing important roles in cancer cell survival and invasion, including in HCC. However, how IMD affects the behavior of HCC cells and the underlying mechanisms have not been fully elucidated. Here, we show that IMD maintains an important homeostatic state by activating the ERK1/2-EGR1 (early growth response 1) signaling cascade, through which HCC cells acquire a highly invasive ability via significantly enhanced filopodia formation. The inhibition of IMD blocks the phosphorylation of ERK1/2, resulting in EGR1 downregulation and endoplasmic reticulum stress (ER) stress, which is evidenced by the upregulation of ER stress marker DDIT3 (DNA damage-inducible transcript 3). The high level of DDIT3 induces HCC cells into an ER-stress related apoptotic pathway. Along with our previous finding that IMD plays critical roles in the vascular remodeling process that improves tumor blood perfusion, IMD may facilitate the acquisition of increased invasive abilities and a survival benefit by HCC cells, and it is easier for HCC cells to obtain blood supply via the vascular remodeling activities of IMD. According to these results, blockade of IMD activity may have therapeutic potential in the treatment of HCC.

## Introduction

Hepatocellular carcinoma (HCC) is the third most common malignant tumor in the world and is characterized by rapid development and a high mortality rate^1–5^. Surgery is currently the most effective treatment, but it is only suitable for patients who are diagnosed early [stage 0 and stage A of Barcelona clinic liver cancer (BCLC)] and have no distant metastasis^4,6^. HCC cells are highly malignant and prone to postoperative recurrence and intrahepatic invasion^7^, and the rate of distant metastasis reaches 40–71.6%^8^.

Local invasion and distant metastasis are the most important biological characteristics of malignant tumors and the major causes of death in cancer patients^9–12^. As one of the most malignant cancer types, HCC is highly invasive and capable of metastasizing to distant organs. Although considerable progress has been made in the area of HCC, the molecular mechanism that affects the ability of HCC cells to migrate, invade and metastasize has not been fully clarified. Thus, further studies on HCC signaling pathways and their regulatory mechanisms in invasion and metastasis will provide new targets and a scientific basis for screening new molecular markers for the early prediction of HCC invasion and metastasis and for seeking more effective therapeutic strategies for HCC.

Intermedin (IMD; or adrenomedullin 2, Adm2) is an endogenous peptide belonging to the calcitonin family. Previous studies on IMD mainly focused on its cardiovascular functions^13–15^. Recent studies have shown that IMD is also associated with tumorigenesis and the prognosis of cancer patients^16–18^. In 2008, Morimoto et al. first reported that IMD expression was upregulated in adrenal tumors^18^. Compared with expression in normal people, IMD in the peripheral blood of breast cancer and prostate cancer patients was significantly increased, and high IMD levels correlated with poor survival outcomes in breast and prostate cancer patients^16,17^. In addition, it was reported that IMD is overexpressed in HCC and regulates cell proliferation and survival^19^. According to these studies, IMD may participate in biological processes such as cell signal transduction through transcriptional and posttranscriptional regulation. The dysfunction and expression of IMD are related to the occurrence and development of HCC, suggesting that IMD may be used as a new molecular marker for tumor diagnosis and prognosis.

In this study, we aimed to explore how IMD affects the behavior of HCC cells, what the underlying mechanism is, and whether blockade of IMD has a therapeutic effect against HCC. Herein, we show that IMD significantly increases the migration and invasion ability of HCC cells by promoting the formation of filopodia. IMD induces the phosphorylation of ERK1/2, which triggers a robust change in gene transcription. Using gene transcriptome sequencing, we identified that EGR1 (early growth response 1) and DDIT3 (DNA damage-inducible transcript 3) were responsible for the effect of IMD. IMD activates an ERK1/2-EGR1 signaling cascade, through which HCC cells acquire highly invasive abilities and a survival benefit. The blockade of IMD inhibited the phosphorylation of ERK1/2, resulting in a significant downregulation of EGR1 and upregulation of DDIT3, which induced HCC cells into an ER-stress related apoptotic pathway. Our study may provide novel insights into the mechanism of HCC cell invasion and metastasis and provide evidence that anti-IMD strategies might have therapeutic effects against HCC.

## Results

### IMD expression was higher in HCC patients with metastasis

**Figure 1:**
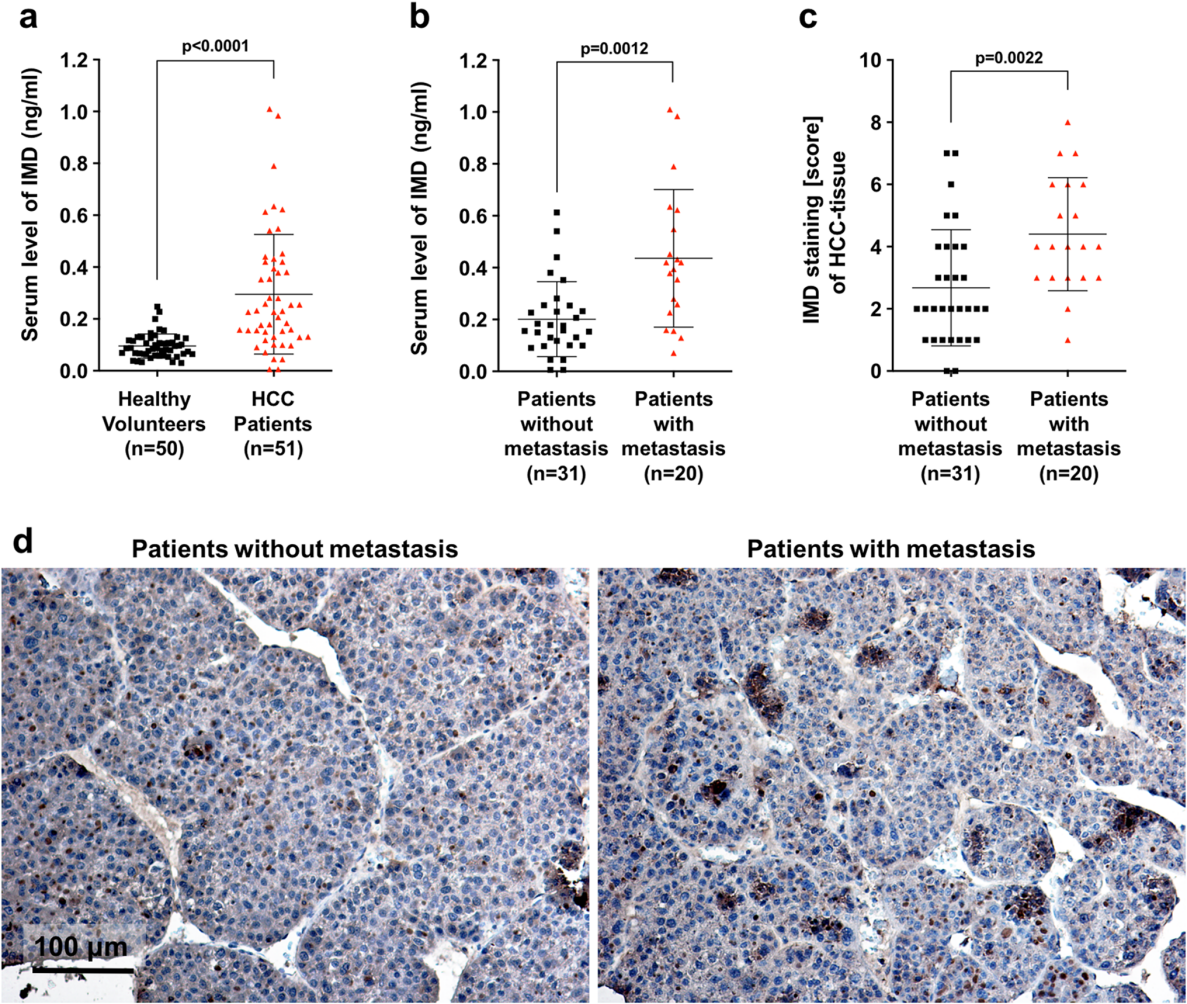
IMD expression was higher in HCC patients with metastasis. (**a**) The IMD levels in healthy volunteers and in patients with HCC were presented as scatter plots with mean ± SD. (**b**) The IMD mRNA levels in HCC patients with or without metastasis. (**c**) The IMD staining scores in HCC patients with or without metastasis. (**d**) Representative images of the IHC staining. Significance was assessed by *unpaired t test with Welch's correction*.

Firstly, we sought to determine whether the expression of IMD was correlated with the pathogenesis of HCC patients. Fifty-one HCC patients and 50 healthy volunteers were enrolled in this study. Serum IMD levels were found significantly increased in HCC patients compared with healthy volunteers (Fig. 1a). In addition, IMD levels in patients with metastasis were significantly higher than in patients without metastasis (Fig. 1b). Immunohistochemistry (IHC) staining showed similar trends in IMD expression in the patients with or without metastasis (Fig. 1c,d).

### IMD expression was higher in more aggressive HCC cells than in less aggressive HCC cells

**Figure 2:**
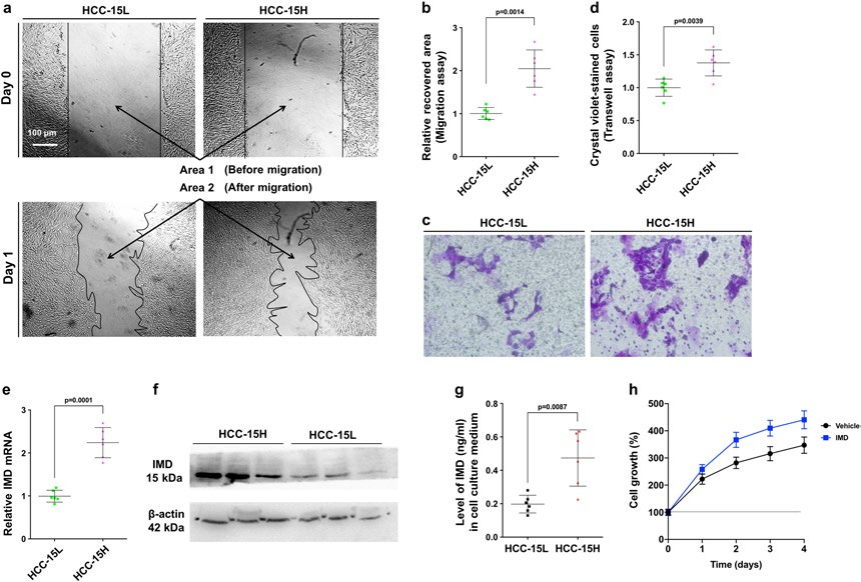
IMD expression was higher in more aggressive HCC cells. (**a**) The HCC-15L and HCC-15H cells were seeded on the 6-well plates. One day after cell scratching, the recovered area was measured by *Area 1* (before cell migration) minus *Area 2* (after cell migration). (**b**) The recovered area (the mean level of the control group was set to 1) was calculated. (**c**) Cells were seeded on the upper chamber of the transwell system. The representative images showed the cells that invaded through the membranes were stained by Crystal Violet. (**d**) The Crystal-Violet-positive cells was counted (relative to the control; the mean level in the control group was set to 1.0; n = 6.). (**e**) The relative IMD mRNA level was measured using Real-time PCR. (**f**) The protein expression level of IMD was measured by Western blot analysis (WB) using the anti-IMD mAb. (**g**) The IMD levels in medium from HCC-15L or HCC-15H culture tested by ELISA. (**h**) 1 × 10^4^ HCC-15L cells were seeded on the 24-well plate and treated with vehicle or IMD for 4 days. The cell number was counted every day from day 0 to day 4, and presented relative to that of day 0. All data were presented as scatter plots with mean ± SD (n = 6). Significance was assessed by *unpaired t test with Welch's correction*.

Primary HCC cells were collected from surgical samples, and two HCC cell lines with high/low invasive ability (named HCC-15H and HCC-15L cells) were screened in severe combined immune deficiency (SCID) mice according to their subcutaneous tumorigenicity and spontaneously metastatic ability. Wound healing assay (Fig. 2a,b) and Transwell assays (Fig. 2c,d) showed that the migration and invasion capacities of HCC-15H cells were significantly higher than those of HCC-15L cells. Consistent with the above clinical data, the mRNA level (Fig. 2e) and protein expression of IMD (Fig. 2f) in HCC-15H cells were significantly higher than that in HCC-15L cells, suggesting that IMD may be closely related to the invasion ability of HCC cells.

The ELISA assay showed that in cell culture medium, IMD secreted by HCC-15H cells was significantly higher than that of HCC-15L cells, indicating that IMD may function in a paracrine/endocrine manner (Fig. 2g). The cells growth assay showed that IMD treatment increased the proliferation of HCC-15L cells (Fig. 2h). Thus, the increased invasive ability may also be due to the cell proliferation. Nevertheless, the time for detecting the migration and invasion was 24 h after IMD treatment, while cell growth assay showed that at the time point of 24 h, the advantage of cell proliferation after IMD treatment was not so significant as the migration experiments. Therefore, at least within 24 h, the effect of IMD on cell migration was likely greater than its effect on cell proliferation.

### IMD promoted the formation of filopodia, which increased HCC cell migration and invasion abilities

**Figure 3:**
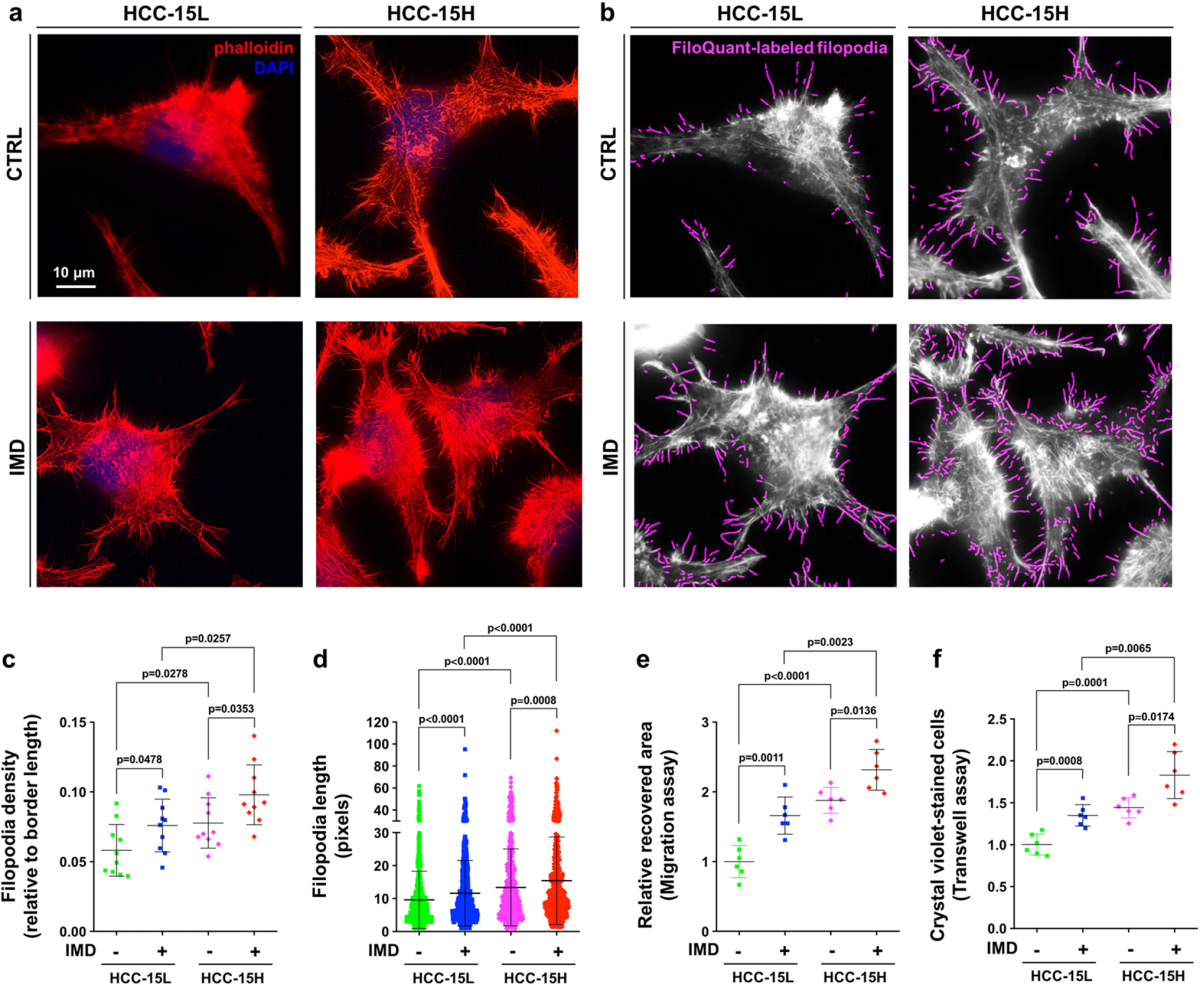
IMD promoted the formation of filopodia, which increased the HCC cell migration and invasion ability. (**a**) HCC-15L and HCC-15H cells treated with or without IMD were stained with AlexaFluo568-phalloidin (red) and DAPI (blue), and observed under 1000 × oil immersion lens. (**b**) The microscopic images were analyzed using FiloQuant. The filopodia were marked with pseudo color. (**c**) The filopodia density (the number of filopodia relative to border length [pixels]) were measured using 10 randomly chosen fields. (**d**) The filopodia length (pixels) were counted using 10 randomly chosen fields. (**e**,**f**) The cell migration and invasion ability were measured using *Wound healing assay* and *Transwell assay* (n = 6). All data were presented as scatter plots with mean ± SD. Significance was assessed by *unpaired t test with Welch's correction* (**c**,**e**,**f**, which passed the normality test) or *Mann–Whitney U test* (**d**, which did not pass the normality test).

Filopodia, finger-like protrusions on the cell surface, are bundled actin-containing plasma membrane protrusions that aid in cancer cell migration and metastasis^20,21^. Despite their broad biological significance, studying these unique structure remains challenging, especially due to the lack of compatible methods to quantify filopodia properties. Herein, we used a freely available ImageJ plugin, FiloQuant, to analyze microscopic images of filopodia^22^. As shown in Fig. 3, the filopodia stained by AlexaFluo568-conjugated phalloidin (Fig. 3a) were labeled and measured using FiloQuant software (Fig. 3b). The quantification results showed that the density and length of filopodia on the surface of HCC-15H cells were significantly higher than those of HCC-15L cells (Fig. 3c,d). In addition, exogenous administration of IMD peptide could further increase the filopodia density and length of both cell types (Fig. 3c,d). The IMD-induced filopodia formation was consistent with the increased migration and invasion abilities of both HCC-15L and HCC-15H cells (Fig. 3e,f, and Supplementary Fig. S1a, b).

### Anti-IMD antibodies markedly inhibited in situ tumor growth and lung metastasis

**Figure 4:**
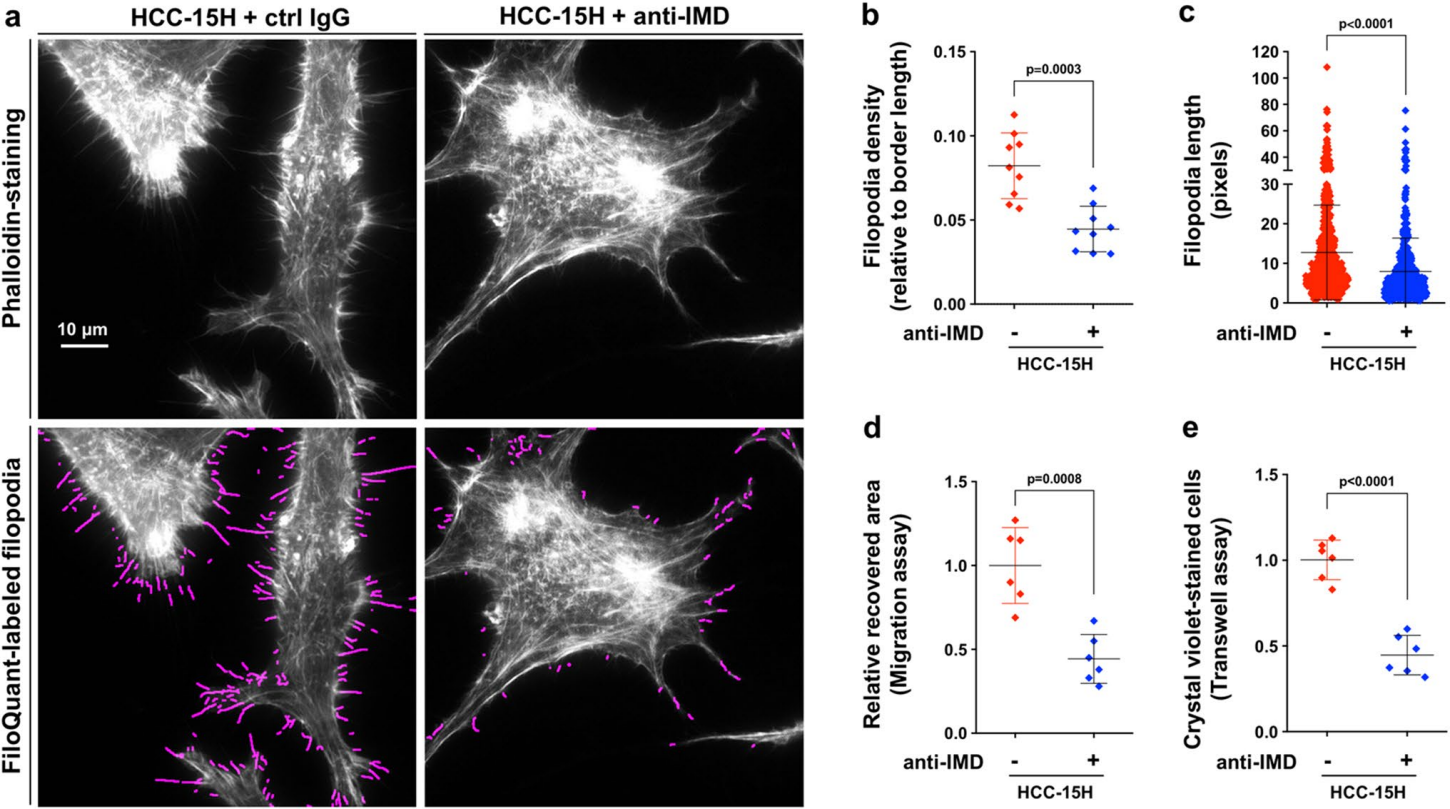
Anti-IMD inhibited the formation of filopodia and invasive ability of HCC cells. (**a**) HCC-15H cells treated with or without anti-IMD were stained with AlexaFluo568-phalloidin and analyzed using FiloQuant. (**b**,**c**) The filopodia density and length were measured using 10 randomly chosen fields. (**d**,**e**) The cell migration and invasion ability were measured using *Wound healing assay* and *Transwell assay* (n = 6). All data were presented as scatter plots with mean ± SD. Significance was assessed by *unpaired t test with Welch's correction* (**b**,**d**,**e**) or *Mann–Whitney u test* (**c**).

Monoclonal antibodies against IMD (anti-IMD mAbs) were prepared for the loss of function studies^23^. Anti-IMD antibodies drastically inhibited the formation of filopodia in highly invasive HCC-15H cells (Fig. 4a–c). Consistently, the migration ability and the invasion ability of HCC-15H cells were also inhibited by anti-IMD antibodies (Fig. 4d,e; and Supplementary Fig. S2a, b).

**Figure 5:**
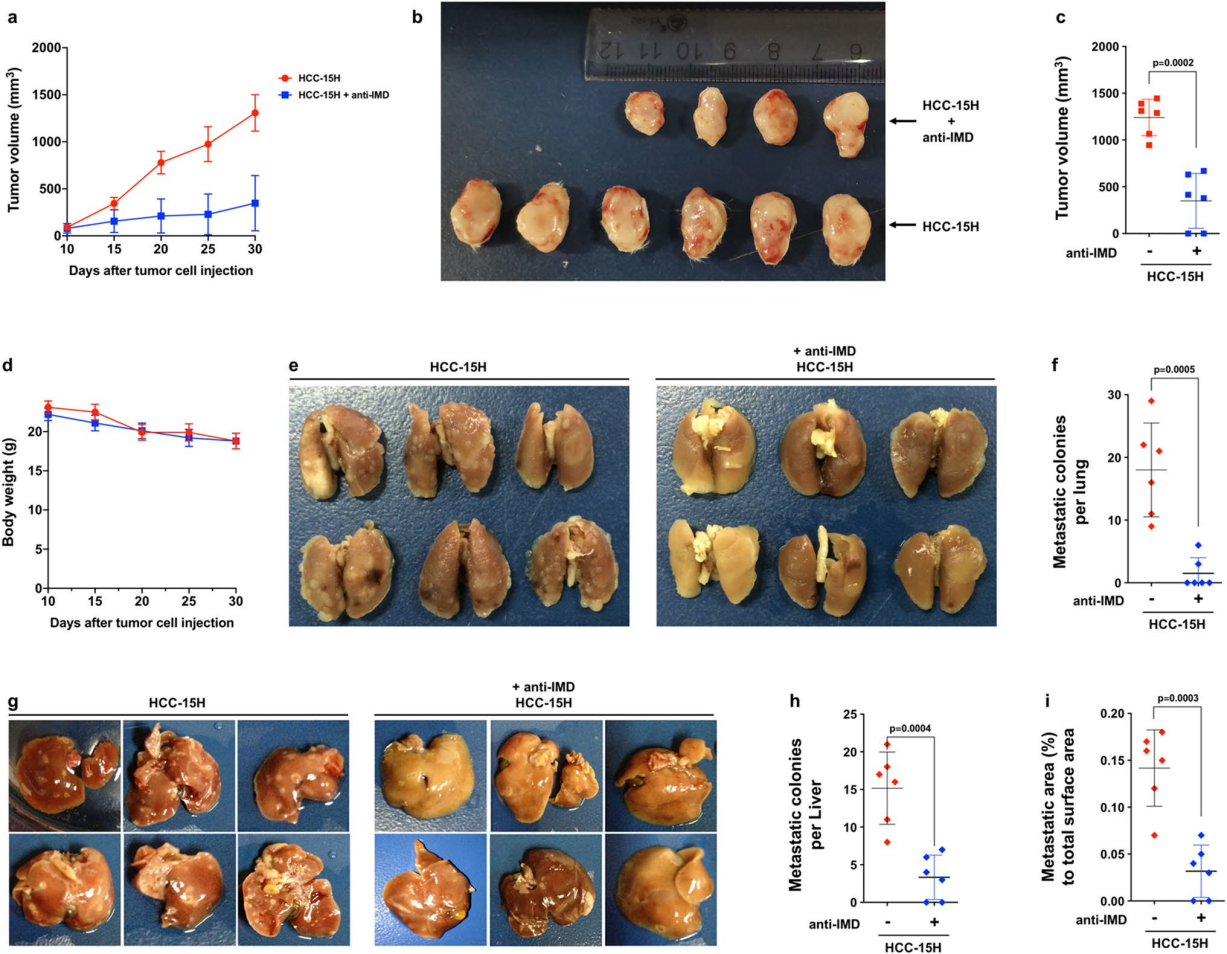
Anti-IMD markedly inhibited the in situ tumor growth and lung metastasis. (**a**) 2.5 × 10^6^ HCC-15H cells were injected subcutaneously into SCID mice. Seven days after tumor inoculation, anti-IMD mAb (5 mg/kg) or control IgG was injected into the mice through tail vein (twice a week, 4 injections). The tumor volumes were measured every 5 days until the biggest tumor reaches approximately 1500 mm^3^. (**b**) After the experiment was terminated, the tumors were removed (**b**), and the final volumes were measured (**c**). (**d**) The bode weight of the tumor-bearing mice were measured every 5 days. (**e**) The lungs from HCC-15H tumor-bearing mice treated with vehicle or the anti-IMD antibody. (**f**) The metastatic colonies in the lungs were counted. (**g**) The liver from HCC-15H tumor-bearing mice treated with vehicle or the anti-IMD antibody. (**h**,**i**) The number and covered area of metastatic colonies in the liver were counted. Data were presented with presented as growth curve (**a**,**d**) or scatter plots with mean ± SD (**c**,**f**). Significance was assessed by *unpaired t test with Welch's correction* (n = 6).

The role of IMD in filopodia formation in HCC cells may favor HCC tumor growth and distant metastasis. To test this, HCC-15H cells were injected subcutaneously into the SCID mice. Seven days after tumor inoculation, anti-IMD antibodies were administered to the tumor-bearing mice (twice a week, 4 injections). At day 30, when the volume of the largest tumor reached approximately 1500 mm^3^, the mice were sacrificed, and the subcutaneous tumors were surgically removed. As shown in Fig. 5a–c, the administration of anti-IMD antibodies markedly inhibited the in situ tumor growth of HCC-15H tumors, and this effect was not due to body-weight loss (Fig. 5d). The lungs from the tumor-bearing mice were also examined. A large number of metastatic colonies were found in the lungs of HCC-15H tumor-bearing mice (Fig. 5e,f). In addition, the intrahepatic metastasis is the most common type of metastasis in HCC cases. We found the subcutaneous inoculation of HCC-15H cells also caused metastatic lesions in the liver (Fig. 5g–i). The administration of anti-IMD antibodies not only significantly reduced the lung metastasis but also decreased the intrahepatic metastasis (Fig. 5e–i). These results suggest that IMD may play important roles in HCC tumor growth and metastasis.

### ERK1/2-EGR1/DDIT3 signaling cascade was responsible for the effect of IMD on HCC cells

**Figure 6:**
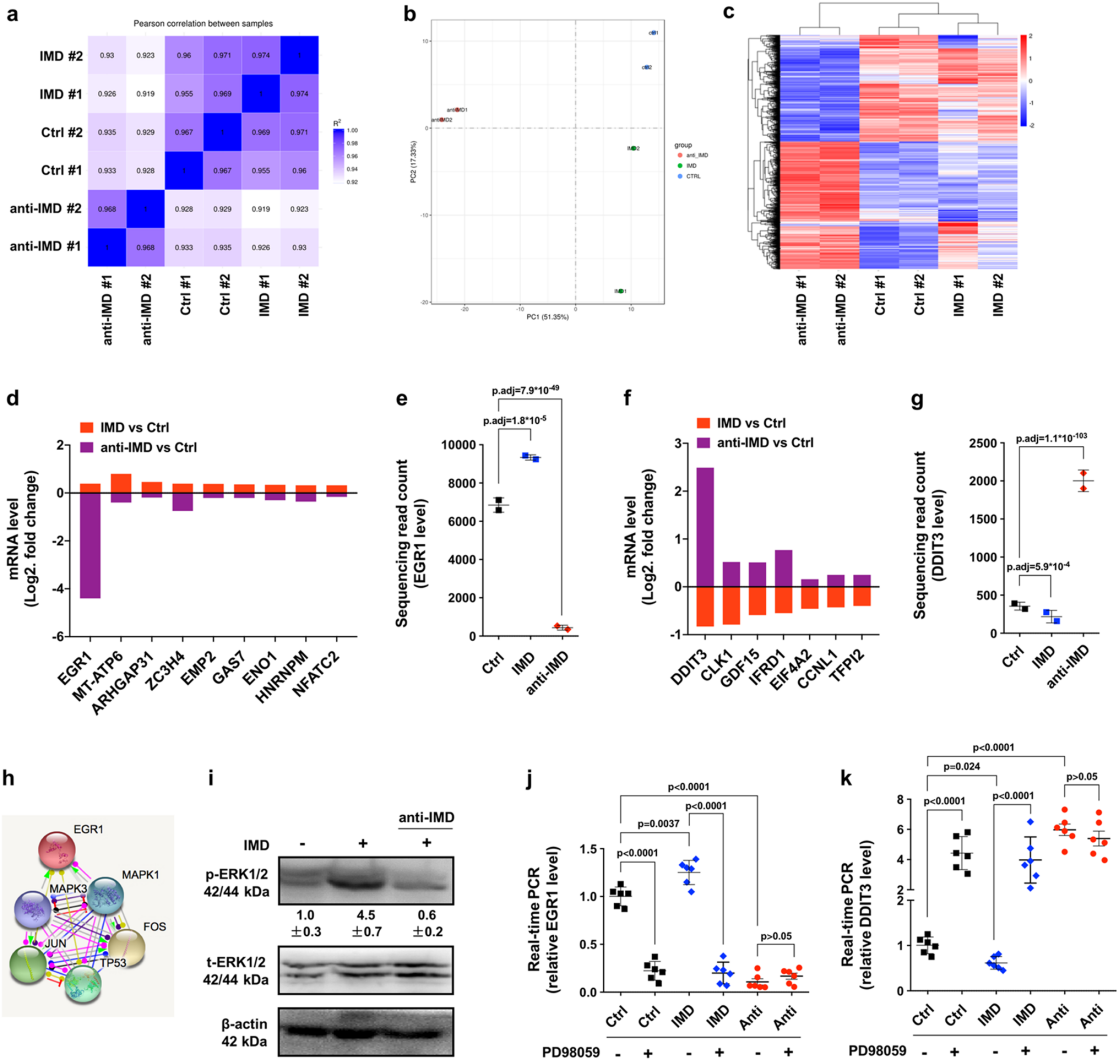
IMD induced phosphorylation of ERK1/2, which significantly increased EGR1 but suppressed DDIT3 transcription. (**a**) The square of Pearson correlation coefficient (R^2^) of the transcriptome sequencing analysis. R^2^ > 0.92 indicates good reliability. (**b**) Principal component analysis (PCA) showed good duplication within groups and significant differences between groups. (**c**) The differentially expressed genes (DEG) heatmap showed IMD or anti-IMD causing significant changes in gene transcription. (**a**–**c** were extracted from the original RNA-Seq report). (**d**) Nine genes were found up-regulated in IMD-treated group, but significantly down-regulated in anti-IMD group. (**e**) The RNA-sequencing read counts of EGR1 in IMD- or anti-IMD-treated group (n = 2). (**f**) Seven genes were found down-regulated in IMD-treated group, but up-regulated in anti-IMD group. (**g**) The RNA-sequencing read counts of DDIT3 in IMD- or anti-IMD-treated group (n = 2). (**h**) The STRING protein interaction analysis of the EGR1 interaction network. (**i**) HCC-15H cells were incubated with IMD or anti-IMD for 10 min and subjected to Western blot assay. The density of the band for p-ERK1/2 (referred to total ERK1/2) is presented relative to that of the control. The mean level in the control group was set to 1.0; n = 3. (**j**,**k**) HCC-15H cells were pre-treated with PD98059; 60 min later, the cells were incubated with IMD or anti-IMD for 10 min, and the mRNA level of EGR1 or DDIT3 were measured using Real-time RT PCR. The mean level in the control group was set to 1.0; n = 6. Data were presented as scatter plots with mean ± SD (**d**,**e**,**f**,**g**,**j**,**k** were generated using the software GraphPad Prism 8.0. https://www.graphpad.com/scientific-software/prism/); and (**h**) was generated using the online tool STRING, https://string-db.org/cgi/input.pl?sessionId=o2YSlODG8A4I&input_page_show_search=on.) Significance was assessed by *unpaired t test with Welch's correction*.

To obtain a more comprehensive understanding of the impact of IMD on HCC cells, we analyzed the gene profiles of HCC-15H cells treated with vehicle, IMD, or anti-IMD antibodies using transcriptome sequencing (RNA-Seq) analysis. Two parallel samples from each group were analyzed, and the correlation coefficients of intragroup and intergroup samples were calculated. The square of the Pearson correlation coefficient (R^2^) in each group was greater than 0.92 (Fig. 6a), indicating good reliability of the experiments and a high similarity of expression patterns between samples. Principal component analysis (PCA) showed that the samples between groups were scattered, and the samples within groups were clustered, indicating good duplication within groups and significant differences between groups (Fig. 6b).

We hypothesized that there may be one or more key factors that serve as downstream effectors for IMD to regulate the behavior of HCC cells. As shown in the DEG (differentially expressed gene) clustering heatmap, IMD or anti-IMD antibodies caused significant changes in gene expression (Fig. 6c). We sought to identify the genes that were significantly upregulated in the IMD-treated group but significantly downregulated in the anti-IMD-antibody-treated group or those that had opposite trends. Nine candidates (EGR1, MT-ATP6, ARHGAP31, ZC3H4, EMP2, GAS7, ENO1, HNRNPM, and NFATC2) that agreed with the former trend were identified. Among them, the change in EGR1 was the most obvious (Fig. 6d). Compared to the level in the vehicle-treated groups, the transcription level of EGR1 in the IMD-treated group increased by approximately 28% (read count: 9,336 vs. 6,846), whereas the level of EGR1 in the anti-IMD-antibody-treated group decreased by approximately 15-fold (read count: 438 vs. 6846) (Fig. 6e). On the other hand, 7 genes (DDIT3, CLK1, GDF15, IFRD1, EIF4A2, CCNL1, and TFPI2) were downregulated in the IMD group but upregulated after anti-IMD antibody treatment (Fig. 6f). Among them, the change in DDIT3 was the most obvious. The level of DDIT3 in the IMD-treated group decreased by approximately 63% (read count: 217 vs. 355), whereas the level of DDIT3 in the anti-IMD-antibody-treated group increased by approximately 5.6-fold (read count: 2001 vs. 355) (Fig. 6g).

EGR1 is a zinc-finger transcription factor that plays important roles in many biological processes^24^. The IHC analysis showed that EGR1 was mainly expressed in the nucleus. IMD slightly increased the expression of EGR1, but anti-IMD drastically reduced the expression of EGR1 in the nucleus (Supplementary Fig. S3). This is consistent with the RNA-Seq results. We used the STRING protein interaction database to analyze the EGR1 interaction network. MAPK1 and MAPK3 (ERK1/2) were shown to be the closest proteins linked to EGR1 and had positive effects on EGR1 (Fig. 6h). Indeed, EGR1 has been reported to be a downstream effector of activated ERK1/2^25–29^. Interestingly, our previous reports have shown that IMD potently induces the phosphorylation of ERK1/2 in endothelial cells^23,30^. We then asked whether IMD induces the phosphorylation of ERK1/2 in HCC cells. Western blot (WB) analysis showed that IMD significantly induced ERK1/2 phosphorylation in HCC-15H cells, and this effect was abrogated by treatment with anti-IMD antibodies (Fig. 6i). To test whether EGR1 serves as a downstream effector of IMD-activated ERK1/2, we used PD98059 to inhibit the phosphorylation of ERK1/2. As shown in Fig. 6h, PD98059 completely blocked the IMD-induced upregulation of EGR1 and decreased EGR1 transcription to the level seen with anti-IMD antibody treatment (Fig. 6j).

DDIT3 is also known as CHOP (CCAAT/enhancer binding proteins [C/EBP] homologous protein), belonging to the C/EBP transcription factor family that regulates a variety of genes involved in a broad range of biological processes, including immune functions, cell differentiation, proliferation, and apoptosis^31^. DDIT3 has also been reported to be related to ERK1/2^32–35^, and blocking ERK1/2 activity caused a significant transcriptional change in DDIT3, similar to the effect of anti-IMD antibodies (Fig. 6k). According to the results, ERK1/2 may serve as a signalling hub that orchestrates the gene regulation of EGR1 and DDIT3, and the expression changes of EGR1/DDIT3 had opposite trends.

**Figure 7:**
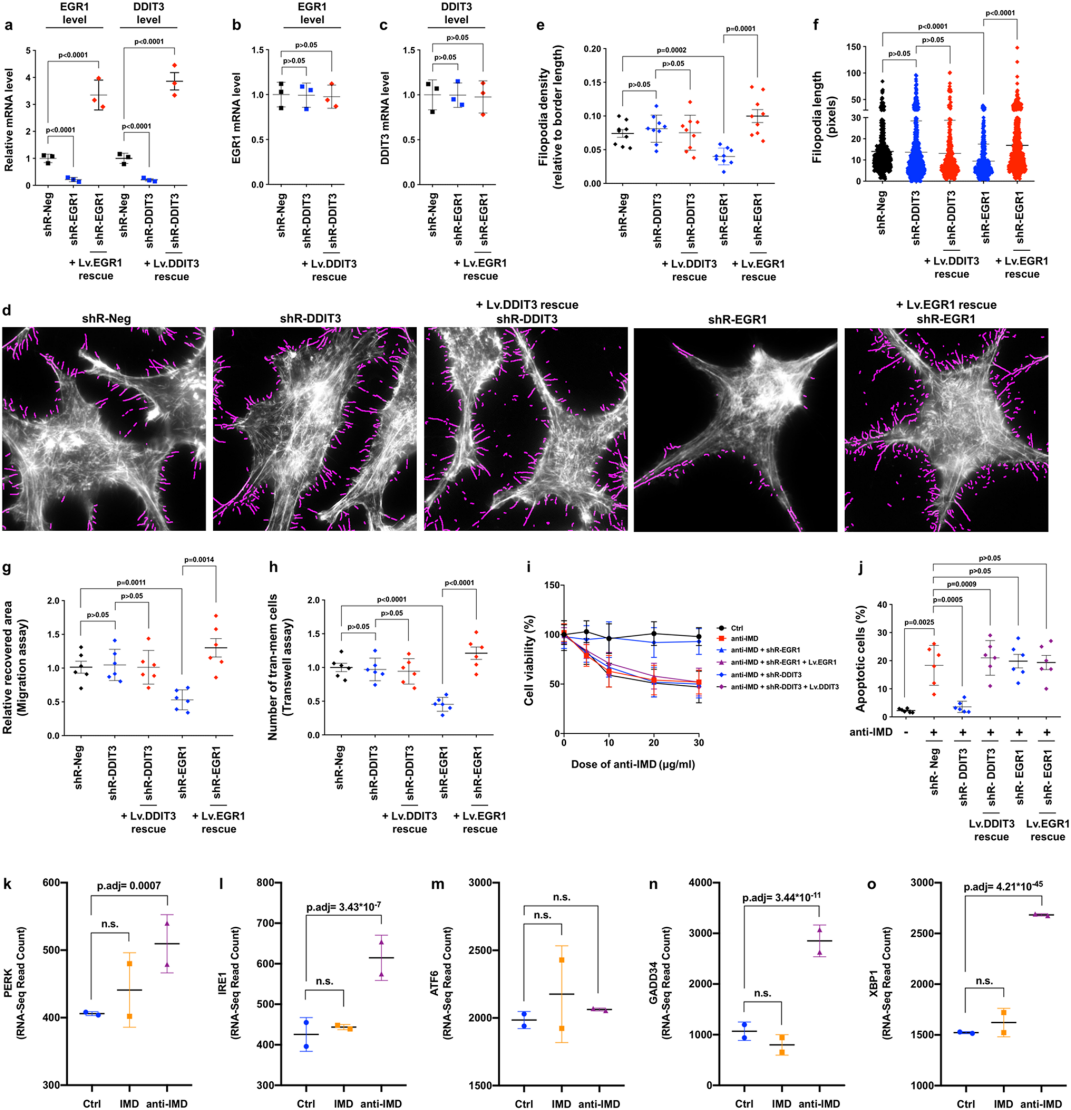
EGR1 and DDIT3 were responsible for the effect of IMD on HCC cells. (**a**) The HCC-15H cells were transfected with shRNAs that target the 3′UTR region of EGR1 or DDIT3 (with or without Lv. EGR1 an Lv. DDIT3 rescuing). After 48 h, the mRNA levels of EGR1 or DDIT3 was measured using Real-time PCR. (**b**,**c**) The mutual effects between EGR1 and DDIT3 by measuring the mRNA level of EGR1 or DDIT3 under the condition of another gene being knockdown or overexpression (n = 3). (**d**) The HCC-15H cells transfected with shRNA-EGR1, shRNA-DDIT3 (with or without Lv. EGR1 an Lv. DDIT3 rescuing) were stained by AlexaFluo568-phalloidin and analyzed using FiloQuant. (**e**,**f**) The filopodia density and length were measured using 10 randomly chosen fields. (**g**,**h**) The cell migration and invasion ability were measured using *Wound healing assay* and *Transwell assay* (n = 6). (**i**,**j**) Before the HCC-15H cells were treated by different dose of anti-IMD, the cells were transfected with shRNA-DDIT3 or shRNA-EGR1 (with or without Lv. EGR1 an Lv. DDIT3 rescuing), and the cell viability (**i**) and apoptosis was measured (**j**). Data were presented as scatter plots with mean ± SD (n = 6). (**k**–**o**) The RNA-sequencing read counts of PERK, IRE1, ATF6, GADD34, XBP1 in IMD- or anti-IMD-treated group (n = 2). Significance was assessed by *unpaired t test with Welch's correction* (**a**,**b**,**c**,**e**,**g**,**h**,**j**), *Mann–Whitney u test* (**f**), and p.adj comparison in the RNA-Seq DEG analysis (**k**–**o**).

To test how EGR1 and DDIT3 affect each other, shRNAs that target the 3′-UTR regions of EGR1 or DDIT3 were transfected into HCC-15H cells using lentiviral vectors, followed by gene-rescue by transfection of an EGR1 construct (Lv. EGR1) or DDIT3 construct (Lv. DDIT3) that did not contain the 3′-UTR region. The gene-silencing and gene-rescue (overexpression) efficiency were verified using semiquantitative real-time PCR analysis (Fig. 7a). We then evaluated the mutual effects between EGR1 and DDIT3 by measuring the mRNA level of EGR1 or DDIT3 when the other gene was knocked down. As shown in Fig. 7b,c, silencing or overexpression of EGR1 or DDIT3 did not affect the transcription of each other.

The shRNA-transfected cells were then stained with AlexaFluo568-phalloidin and subjected to FiloQuant analysis (Fig. 7d). DDIT3 knockdown did not affect the formation of filopodia. However, EGR1 knockdown drastically reduced the density and length of filopodia on the cell surface (Fig. 7d–f), and significantly inhibited HCC cell migration and invasion (Fig. 7g,h, Supplementary Fig. S4). To exclude possible off-target effects, the cells transfected with shR-EGR1 or shR-DDIT3 were rescued by transfection of Lv. EGR1 or Lv. DDIT3. The EGR1 gene overexpression significantly promoted filopodia formation, cell migration and invasion, whereas DDIT3 overexpression did not affect these functions (Fig. 7d–h). According to these results, EGR1, but not DDIT3, may be responsible for the inhibitory effects of anti-IMD antibodies on HCC cell filopodia formation and invasion.

The cell viability assay showed that anti-IMD antibodies dose-dependently induced HCC cell death in vitro (Fig. 7i), which may be because anti-IMD antibodies induced significant HCC cell apoptosis (Fig. 7j). DDIT3 has been found to be the key effector that mediates the ER stress-induced apoptotic pathway^31,36–38^. Indeed, DDIT3 knockdown nearly completely abrogated the anti-IMD-antibody-induced apoptosis and killing activity (Fig. 7i,j).

To exclude possible off-target effects, the HCC-15H cells transfected with shR-DDIT3 were rescued by transfection with a DDIT3 construct (Lv. DDIT3) that did not contain the 3′-UTR region. The DDIT3 gene rescue restored the ability of anti-IMD antibodies to induce HCC cell apoptosis and death (Fig. 7i,j). However, the EGR1 knockdown or overexpression did not affect the HCC cell viability and apoptosis (Fig. 7i,j). Thus, the drastic upregulation of DDIT3 induced by anti-IMD antibodies may be responsible for the inhibitory effects of anti-IMD antibodies on HCC tumor growth.

Activation of the unfolded protein response (UPR) depends on three ER stress sensor proteins, that is, Inositol requiring enzyme 1 (IRE1), Protein kinase RNA (PKR)-like ER kinase (PERK), and activating transcription factor 6 (ATF6). The RNA-Seq showed that the anti-IMD antibody significantly upregulated the expression of IRE1 and PERK, but no ATF6. The treatment of IMD did not affect the expression of the 3 genes may be due to that the IMD-induced of ERK1/2 phosphorylation increases cell growth and invasive ability, but it will not induce UPR, therefore it will not affect the expression of the ER-stress sensor proteins (Fig. 7k–m). In addition, XBP1 (X-box binding protein 1), the downstream UPR target gene of IRE1, and GADD34 (growth arrest and DNA damage-inducible 34) and DDIT3, the downstream UPR targets of PERK, were also significantly increased after anti-IMD treatment (Fig. 7n,o). According to these results, the blockade of IMD may induce ER-stress via IRE1 and PERK, the two canonic sensors of UPR.

## Discussion

Previous studies have suggested that IMD may play important roles in cancer cell survival and invasion^16–19^, including in HCC^19^. However, how IMD affects the behavior of HCC cells and the underlying mechanisms have not been clearly elucidated. In this study, we found that IMD maintained an important homeostatic state by activating the ERK1/2-EGR1 signaling cascade, through which HCC cells acquire highly invasive abilities and a survival benefit. The inhibition of IMD using anti-IMD antibodies blocked the phosphorylation of ERK1/2, inducing ER stress and significant upregulation of DDIT3, which suppressed EGR1 transcription and induced apoptosis (Supplementary Fig. S5).

Previous studies on IMD mainly focused on its cardiovascular activity^14,15^. The relationship between IMD and cancer was first reported in 2008, showing that IMD expression was increased in adrenal tumors^18^. IMD level in peripheral blood was also reported to be associated with the prognosis of breast cancer^17^ and prostate cancer^16^. Interestingly, Guo and colleagues have reported that IMD is overexpressed in HCC and regulates HCC cell proliferation and survival^19^. They found that blocking IMD signaling inhibited HCC growth in a dose-dependent manner by inducing apoptosis, findings that are consistent with our results. However, they reported that the IMD-blockade-induced apoptosis was mediated by the Gli1-Blc2 pathway, whereas our results suggest that anti-IMD antibodies may cause homeostatic disturbance and ER stress, which triggers apoptosis via accumulation of DDIT3. This difference may be because they mainly used the HCC cell lines SK-Hep-1 and SNU-398, whereas we used primary HCC cells (HCC-15L and HCC-15H, from surgical samples) as our major research models.

Activation of the UPR depends on IRE1, PERK, and ATF6, the three ER stress sensor proteins. We found that the anti-IMD antibody upregulated the expression of IRE1 and PERK, but did not affect the expression of ATF6. IRE1 belongs to type I transmembrane proteins, and is the only ER stress sensor present in all eukaryotes and therefore reflects the most ancient and most conserved branch of the UPR^39^. PERK is also a type I transmembrane protein that partially resembles IRE1^40^. Unlike Ire1 and PERK, ATF6 is a type II transmembrane protein with a carboxy-terminal stress-sensing lumenal domain^41^. The difference may be the reason why the two kinds of sensors react differently to IMD. On the other hand, the treatment of IMD did not seem to affect the expression of the ER stress sensor genes, which may be due to that the IMD-induced of ERK1/2 phosphorylation increases cell growth and invasive ability, but it will not induce UPR, therefore it will not affect the expression of the ER-stress sensor proteins.

The published literatures have shown that IMD can induce ERK1/2 phosphorylation in multiple types of cells^30,42,43^. IMD shares the receptor CRLR with other family members, such as CGRP and ADM^44–46^. According to our previous researches, IMD induces ERK1/2 phosphorylation via a Src-signaling cascade by interaction with its receptor CRLR (calcitonin receptor-like receptor). We found that IMD induced the formation of a signaling complex containing CRLR and Src and promoted this complex internalizing into cytoplasm in a clathrin-dependent manner to activate downstream ERK1/2^47^. IMD is likely to induce ERK1/2 phosphorylation in tumor cells through the same signaling pathway because CRLR and Src are commonly expressed in tumor cells and normal cells. During the research on IMD, we have found that IMD can activate ERK1/2 by inducing Src phosphorylation in more than 20 kinds of cells (including tumor cells such as liver cancer cells, breast cancer cells, glioma cells; and normal cells such as endothelial cells, fibroblasts, smooth muscle cells, etc.). According to these results, the IMD-ERK1/2 cascade may be considered to be an established signaling pathway.

Previous studies have not mentioned the role of IMD in cancer cell motility or invasive abilities. Here, we show that IMD increases HCC cell migration and invasion abilities by promoting the formation of filopodia. Although IMD was reported to be involved in the regulation of RhoA^48^, Rac1^49^, and myosin^50^, which are related to cell contraction and deformation, we did not find proof that IMD affects the expression of RhoA, Rac1 or myosin, at least in the circumstances of this study. Instead, our study suggests that the induction of EGR1 transcription induced by ERK1/2 activation may be responsible for the IMD-induced filopodia formation and cell migration.

Our data suggest that IMD is not only involved in HCC cell invasion and metastasis but also activates a signaling pathway that makes HCC cells prone to survival. In addition, we have previously shown that IMD plays a critical role in the vascular remodeling process that improves tumor blood perfusion^30^. Thus, the high level of IMD may facilitate the acquisition of increased invasive abilities and a survival benefit by HCC cells, and it is easier for HCC cells to obtain blood supply via the vascular remodeling activities of IMD. According to these results, blockade of IMD activity may have therapeutic potential in the treatment of HCC.

## Experimental procedures

### Cells and culture conditions

The primary HCC tumors were collected from the surgical samples. The resected tumor tissues from HCC patients at West China Hospital were mechanically digested and temporarily cultured in vitro. The cells were then collected and mixed 1:1 with *Matrigel* (Corning) and injected subcutaneously into SCID mice. Once established, the subcutaneous tumors were collected again and re-established on another batch of SCID mice. According to the subcutaneous tumorigenicity and the metastatic ability (spontaneously metastasizes to the lungs or not), two cell lines (HCC-15L & HCC-15H) were screened out for this study. HCC-15L cell was able to form subcutaneous tumor and grew at a relatively medium speed, but unable to metastasize to the lungs; on the other hand, HCC-15H cell grew faster than HCC-15L, and was able to spontaneously metastasize to the lungs (100% lung metastasis). The cells were established in June 2018, authenticated using PowerPlex 18D System (Promega Corporation), and the last time these cells were tested was June 2019. For in vitro studies, the tumor tissues were digested into single cell suspensions through enzymatic digestion methods. The cells were routinely grown in DMEM supplemented with 10% fetal bovine serum at 37 °C and 5% CO_2_.

### Antibodies and reagents

Antibody for Ki-67 (Cat. RM-9106) was from Thermo Scientific; antibodies for total-ERK1/2 (#4695) and p-ERK1/2 (#4370) were from Cell Signaling Technology; AlexaFluo568-conjugated phalloidin (A12380) was from Invitrogen, Thermo Fisher; PD98059 was from Sigma-Aldrich, Merck. The shRNA targeting 3′-UTR of EGR1 (Clone ID: V2LHS_262011) and DDIT3 (Clone ID: V3LHS_646287) were from Dharmacon. The IMD mature peptide was synthesized and purchased from Shinegene, and the anti-IMD mAb (clone number: 1106CT1.2.1; isotype: IgG2b) was customized and purified by Abgent Co^30^.

### The monoclonal antibody to IMD

The monoclonal antibody that recognizes the epitope of mouse IMD (CRPAGRRDSAPVDPSSPHSY) was generated, as described in^23^. In brief, 6 subclones (929CT5.1.1, 929CT19.2.1, 1072CT2.1.1, 1072CT2.1.2, 1106CT1.2.1, 1106CT2.2.1) that were able to recognize the peptide-KLH antigen were screened after immunization (ELISA assay OD 450 nm, > 1/4000). The isotypes of 1072CT2.1.1 and 1072CT2.1.2 are IgG1, and 1106CT1.2.1 and 1106CT2.2.1 are IgG2b, which are appropriate subtypes to block the activity of target protein in mouse model. Among them, 1106CT1.2.1 showed highest binding capacity to mouse-IMD. In addition, 1106CT1.2.1 was also able to recognize human-IMD, as evidence by the binding capacity assay of the 1106CT1.2.1 to mouse-IMD and human-IMD at the concentrations of 1/500, 1/1000, 1/2000, 1/4000, 1/8000, 1/16,000, 1/32,000, 1/64,000, 1/128,000, 1/256,000.

### Wound healing assay

Cells were seeded on the 6-well plate and cultured until confluent. A straight scratch was made using a pipette tip under an angle approximately 30°, simulating a wound. IMD or anti-IMD were added into the medium as indicated in the figure legends. One day after scratching, the recovered area was determined by *Area 1* (before scratching) minus *Area 2* (24 h after scratching). The experiment was performed in duplicate wells and repeated three times independently.

### Transwell assay

The Transwell assay has been described previously^51^. In brief, 5000 cells were seeded on the Matrigel-coated upper chamber (transwell inserts, pore size: 3.0 μm, Millipore) of the 24-well plates and subsequently incubated at 37 °C, 5% CO2. Following incubation for 24 h, transmigrated cells on the lower surface of the membrane were stained with CFSE, fixed with 4% PFA. The total number of migrated cells was counted under microscope. The experiment was performed in duplicate wells and repeated three times independently.

### Fluorescent microscopy and filopodia quantification

Cells cultured on coverslip were fixed with 4% PFA, and stained with AlexaFluo568-conjugated phalloidin (1 μg/ml) and DAPI (10 μg/ml). The cells were observed under 1000 × oil immersion lens, and the fluorescent images were acquired using a Zeiss Z2 microscope. The filopodia on cell surface were analysed using the software FiloQuant, which was developed as a plugin for the freely available software ImageJ. FiloQuant is a tool for automated detection and quantification of filopodia properties such as length and density.

The Fiji distribution of ImageJ was recommended. To run FiloQuant in Fiji, the following dependencies need to be installed:

1. Enhanced Local Contrast (CLAHE.class; http://imagej.net/Enhance_Local_Contrast_(CLAHE))
2. Skeletonize3D.jar (http://imagej.net/Skeletonize3D),
3. AnalyzeSkeleton.jar (http://imagej.net/AnalyzeSkeleton)
4. Temporal-Color Code (http://imagej.net/Temporal-Color_Code).

The single image analysis of FiloQuant contains step-by-step user validation of the various processing steps to achieve optimal settings for filopodia detection: (1) choose the region of interest; (2) brightness/contrast adjustment; (3) parameters to detect the cell edge; (4) validation of filopodia-free cell edge detection; (5) parameters to detect filopodia; (6) validation of filopodia detection. The FiloQuant will label the filopodia with pseudo color. The filopodia number, the length (pixels) of each filopodia, and the cell edge length (pixels) were automatically output to an Excel file. The filopodia density (the number of filopodia relative to border length [pixels]) and the filopodia length (pixels) were counted using 10 randomly chosen fields (n = 10).

### Western blot (WB) analysis

The method to perform WB assay has been described previously^23^. In brief, Cell extracts were separated by SDS-PAGE and electro-transferred onto polyvinylidene fluoride membranes, and blocked in 5% nonfat milk in Tris-buffered saline/0.01% Tween 20 for 2 h. Blots were incubated at 4 °C in Tris-buffered saline with primary antibody (dilution according to the manufacturer’s instruction), followed by 1 h incubation with horseradish peroxidase-conjugated secondary antibody and detected by a chemiluminescence kit (Millipore, Cat. WBKLS0100).

### Tumor study

All animal experiments were approved by the Animal Ethics Committee of Sichuan University and performed according to institutional and national guidelines. The NOD-SCID female mice (6 weeks, 20-25 g, housed in specific pathogen-free [SPF] conditions) were used in this study. Mice were injected subcutaneously with 2.5 × 10^6^ cells into the shaved right flank. The tumor volume was measure every 5 days after cell injection. The tumor volume was determined by the following formula: Volume (mm^3^) = 1/2 × length (mm) × width (mm) × width (mm). The body weights of the tumor-bearing mice were measure every 5 days accordingly. At the end point when the largest tumor reached approximately 1500 mm^3^ according to the ethical standards for animal welfare, mice were anesthetized and euthanized, and the subcutaneous tumors and the lungs were surgically removed. The metastatic colonies on the surface of lungs were counted under stereoscopic microscope.

### Annexin V/PI apoptotic assay

The Annexin V/PI assay was performed according to the protocol of the KIT supplier (BD bioscience). In brief, 1 × 10^6^ cells were incubated 4–6 h at 37 °C prior to the experiment. Prepare the solution of FITC Annexin V, Propidium Iodide (PI), and 10 × Annexin V Binding Buffer, and stain the cells following the manufacturer’s instruction. Wash cells twice with cold PBS and then re-suspend cells in 1 × Binding Buffer at a concentration of 1 × 10^6^ cells/ml. Transfer 100 μl of the solution (1 × 10^5^ cells) to a 5 ml culture tube. Add 5 μl of FITC Annexin V and 5 μl PI, and gently vortex the cells and incubate for 15 min at RT (25 °C) in the dark. Add 400 μl of 1 × Binding Buffer to each tube. The cells were subjected into Flow cytometry analysis within 1 h.

### Transcriptome sequencing analysis (RNA-Seq)

#### Inter-sample correlation

The correlation of gene expression levels among samples is an important index to test the reliability of experiments and the rationality of sample selection. The closer the correlation coefficient is to 1, the higher the similarity of expression patterns between samples is. The square of Pearson correlation coefficient (R^2^) between biologically repeated samples is usually required be at least greater than 0.8, otherwise we need to make appropriate explanations for the samples, or re-experiment. The R^2^ be greater than 0.92 indicates ideal sampling and experimental conditions. According to the FPKM values of all genes in each sample, the correlation coefficients of intra-group and inter-group samples were calculated, and the thermal maps were drawn to visually show the differences between groups and the repetition of intra-group samples. The higher the correlation coefficient between samples, the closer the expression pattern is. The R2 correlation heat map in this study was shown in Fig. 5a.

#### Principal component analysis

Principal component analysis (PCA) is used to evaluate the differences between groups and sample duplication within groups. PCA uses linear algebraic method to reduce the dimension of genetic variables and extract principal components. We performed PCA analysis of gene expression values (FPKM) in all samples, as shown in Fig. 5b. Ideally, in a PCA diagram, the samples between groups should be scattered and the samples within groups should be clustered.

#### Differential gene clustering analysis

More than two groups of experiments can cluster differentially expressed genes, which may have common functions or participate in metabolic and signaling pathways. Genes with similar expression patterns will be clustered together. The color in each grid reflects not the gene expression value, but the values obtained by homogenizing the rows of the expressed data. Therefore, the color in the heatmap can only be compared horizontally (the expression of the same gene in different samples), but not vertically (the expression of different genes in the same sample). When compared horizontally, red indicates high gene expression and blue indicates low gene expression. The result file contains both inter-group clustering and inter-sample clustering, as shown in Fig. 5c.

### Human study

#### All methods were carried out in accordance with relevant guidelines and regulations

The observational study using the human data for prospective measurements of IMD levels was approved by the ethics committee of West China Hospital, Sichuan University. The method to analyse the human data has been described previously^47^. In brief, 50 healthy volunteers and 51 patients with newly diagnosed HCC were enrolled in this study from the Department of Liver and Vascular Surgery of the West China Hospital, Sichuan University. The diagnosis of HCC included a combination of pathological examination, ultrasound and clinical manifestations. Identification of the stage of tumor is based on the TNM (tumour–node–metastasis) classification system promulgated by the American Joint Committee on Cancer and International Union Against Cancer in 2003. Patients who had other type of cancers were excluded. All subjects were genetically unrelated ethnic Han Chinese from Chengdu City and surrounding regions. The volunteers were not blood relatives of the patients or each other and were frequency-matched with the cases by age at enrollment, sex and residential area. Informed consents were obtained according to the Declaration of Helsinki. After blood collection, serum was separated by centrifugation (2000*g*, 20 min, 4 °C) and stored at − 80 °C until ELISA detection. IMD levels in human serum samples were measured using an ELISA kit (Phoenix Pharmaceuticals, Beijing, China). Serum IMD levels are presented as scatter plots showing the mean ± SD.

### Statistics

The statistical power was calculated to determine the n-number of each group. In the animal studies, no randomization was applied because all mice (NOD-SCID mice) used in this study were genetically defined, inbred mice. When comparing two groups for which a Gaussian distribution was assumed, the unpaired, 2-tailed parametric t test with Welch’s correction was used; when a Gaussian distribution was not assumed, the unpaired, 2-tailed nonparametric Mann–Whitney U test was used. A *p* value < 0.05 was considered statistically significant.

## Supplementary information

Supplementary Figures.
