# Supplementary material for: Intermedin facilitates hepatocellular carcinoma cell survival and invasion via ERK1/2-EGR1/DDIT3 signaling cascade
Source: Sci Rep. 2021 Jan 12;11:488. doi: 10.1038/s41598-020-80066-x (PMC7803743; doi:10.1038/s41598-020-80066-x)
Supplement: Supplementary file 1 — Supplementary Figures. [file 41598_2020_80066_MOESM1_ESM.docx]

**Title: Intermedin facilitates hepatocellular carcinoma cell survival and invasion via ERK1/2-EGR1/DDIT3 signaling cascade**

**Authors: Fei Xiao** M.D.**^#,1^, Hongyu Li** M.D.^#^**^,2^, Zhongxue Feng** M.S ^#^**^, 4^, Luping Huang** M.S. **^#,4^, Lingmiao Kong** M.S. **^4^, Min Li** M.S **^4^, Denian Wang** M.D.**^4^, Fei Liu** M.D. **^3^, Zhijun Zhu** M.D. ^*,^**^2^, Yong’gang Wei** M.D. ^*^**^,3^** **Wei Zhang** Ph.D.^*,^**^4^**

# The authors contributed equally to this work

* Correspondence should be addressed to: Wei Zhang: zhangwei197610@163.com (or to Yong’gang Wei: yourwyg@163.com, or to Zhijun Zhu: Zhu-zhijun@Outlook.com)

1 Department of Intensive Care Unit of Gynecology and Obstetrics, West China Second University Hospital, Sichuan University

2 Liver transplantation center, Beijing Friendship Hospital, Capital Medical University

3 Department of Liver Surgery, West China Hospital, Sichuan University

4 Department of Critical Care Medicine, State Key Laboratory of Biotherapy and Cancer Center, West China Hospital, Sichuan University and Collaborative Innovation Center of Biotherapy

Address: No. 1, Ke Yuan 4th Road, Gao Peng Street, Chengdu, Sichuan, 610041, P. R. China

TEL: 86 (028) 8516-4093

FAX: 86 (028) 8516-4092

**Supplementary Figures**


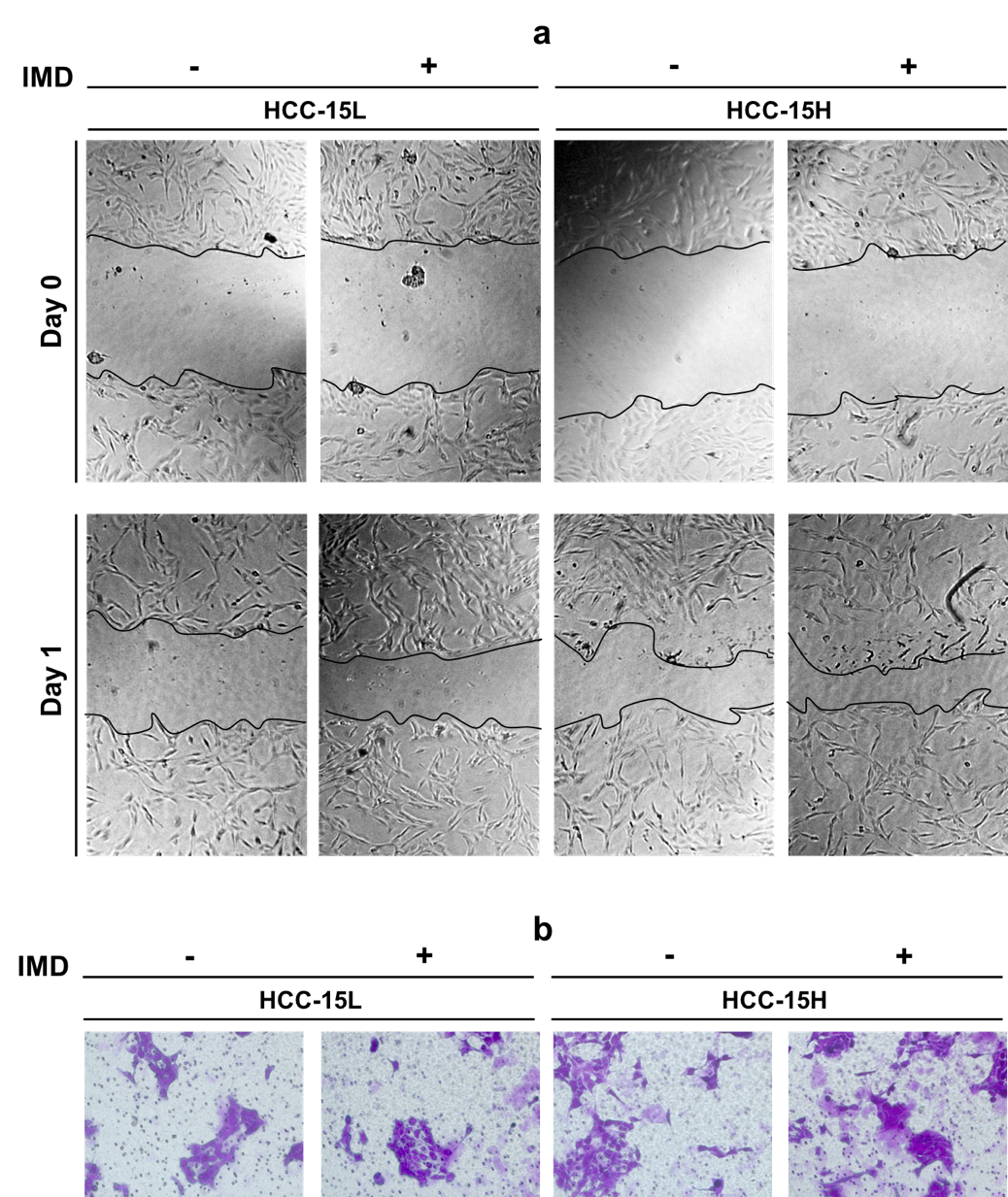


**Supplementary Fig S1. (a)** HCC-15L and HCC-15H cells treated with or without IMD and were seeded on the 6-well plates. One day after cell scratching, the recovered area was measured by *Area 1* (before cell migration) minus *Area 2* (after cell migration). (**b**) The cells were seeded on the upper chamber of the transwell system. The representative images showed the cells that invaded through the membranes were stained by Crystal Violet.


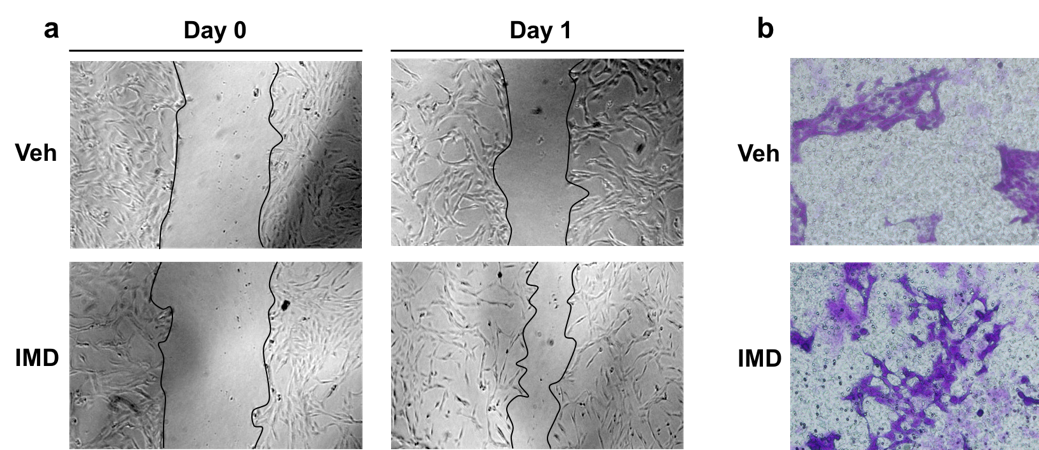


**Supplementary Fig S2. (a)** HCC-15H cells treated with vehicle or anti-IMD monoclonal antibodies were seeded on the 6-well plates. One day after cell scratching, the recovered area was measured by *Area 1* (before cell migration) minus *Area 2* (after cell migration). (**b**) The cells were seeded on the upper chamber of the transwell system. The representative images showed the cells that invaded through the membranes were stained by Crystal Violet.


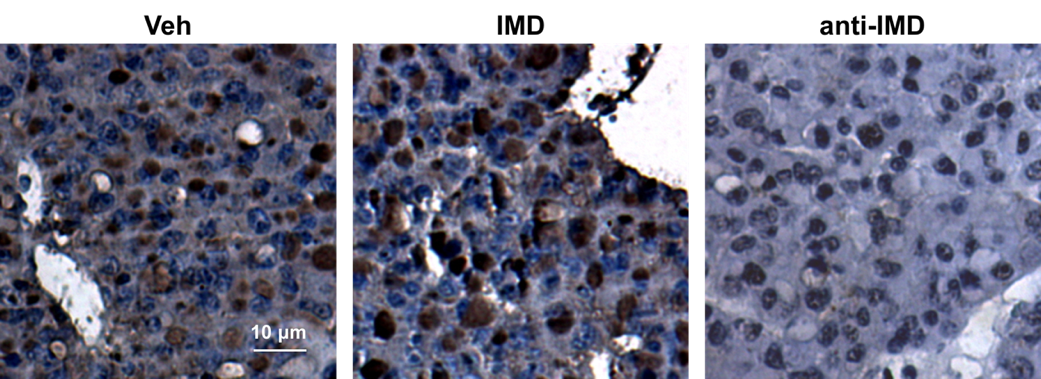


**Supplementary Fig S3.** IHC-staining of EGR1 in the HCC-15H cells treated with IMD or anti-IMD.


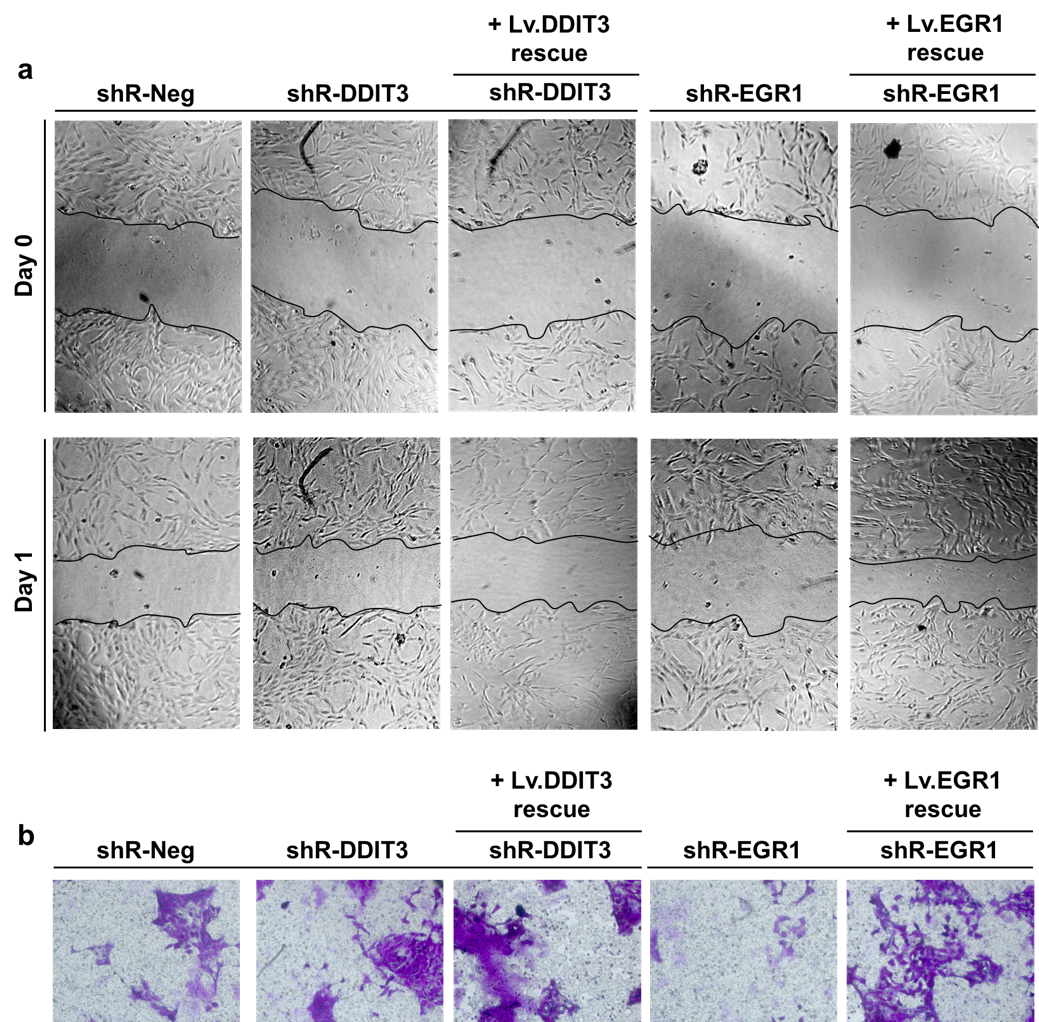


**Supplementary Fig S4. (a)** The HCC-15H cells were transfected with shRNA-Negative, shRNA-EGR1 (with or without the rescue using Lv. EGR1), and shRNA-DDIT3 (with or without the rescue using Lv. DDIT3), and seeded on the 6-well plates. One day after cell scratching, the recovered area was measured by *Area 1* (before cell migration) minus *Area 2* (after cell migration). (**b**) The cells were seeded on the upper chamber of the transwell system. The representative images showed the cells that invaded through the membranes were stained by Crystal Violet.


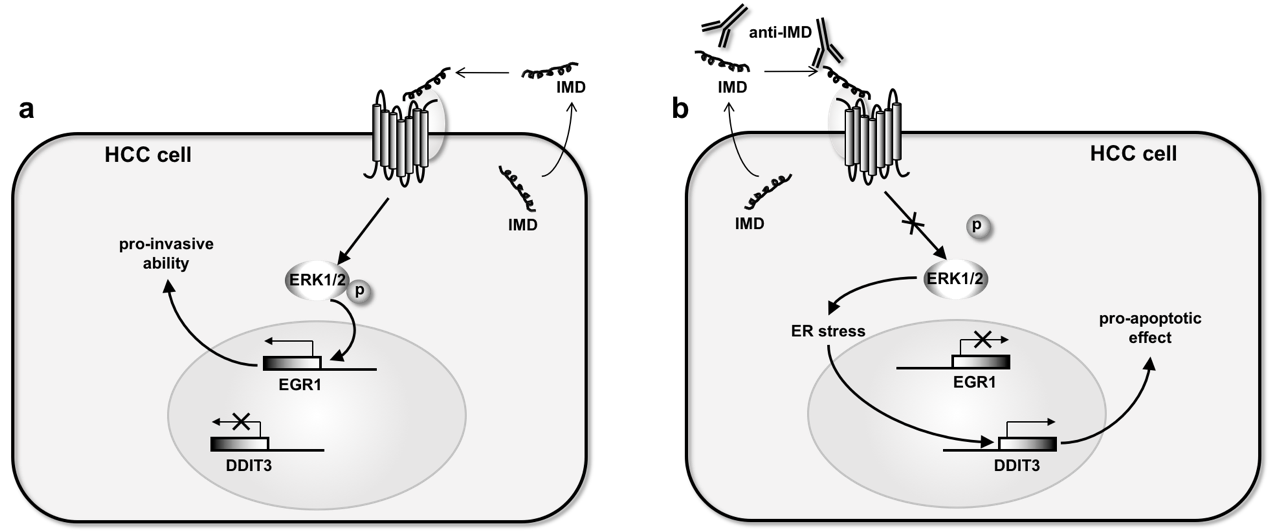


**Supplementary Fig S5.** The schematic diagram of the IMD-manipulated HCC cells: (**a**) In the steady state, HCC cells express high level of IMD, which functions in an autocrine/paracrine manner and induces the phosphorylation of ERK1/2. The IMD-induced ERK1/2 activation promotes EGR1 transcription, which induces an invasive phenotype of the HCC cells. Under this circumstance, the DDIT3 gene is in an inactive state and does not trigger downstream pro-apoptotic effects. (**b**) On the other hand, when the IMD peptides are neutralized by anti-IMD antibodies, the IMD-induced ERK1/2 phosphorylation is blocked, resulting in inhibited EGR1 transcription and homeostatic disturbance, which triggers DDIT3 transcription and the ER stress-induced apoptotic pathway.


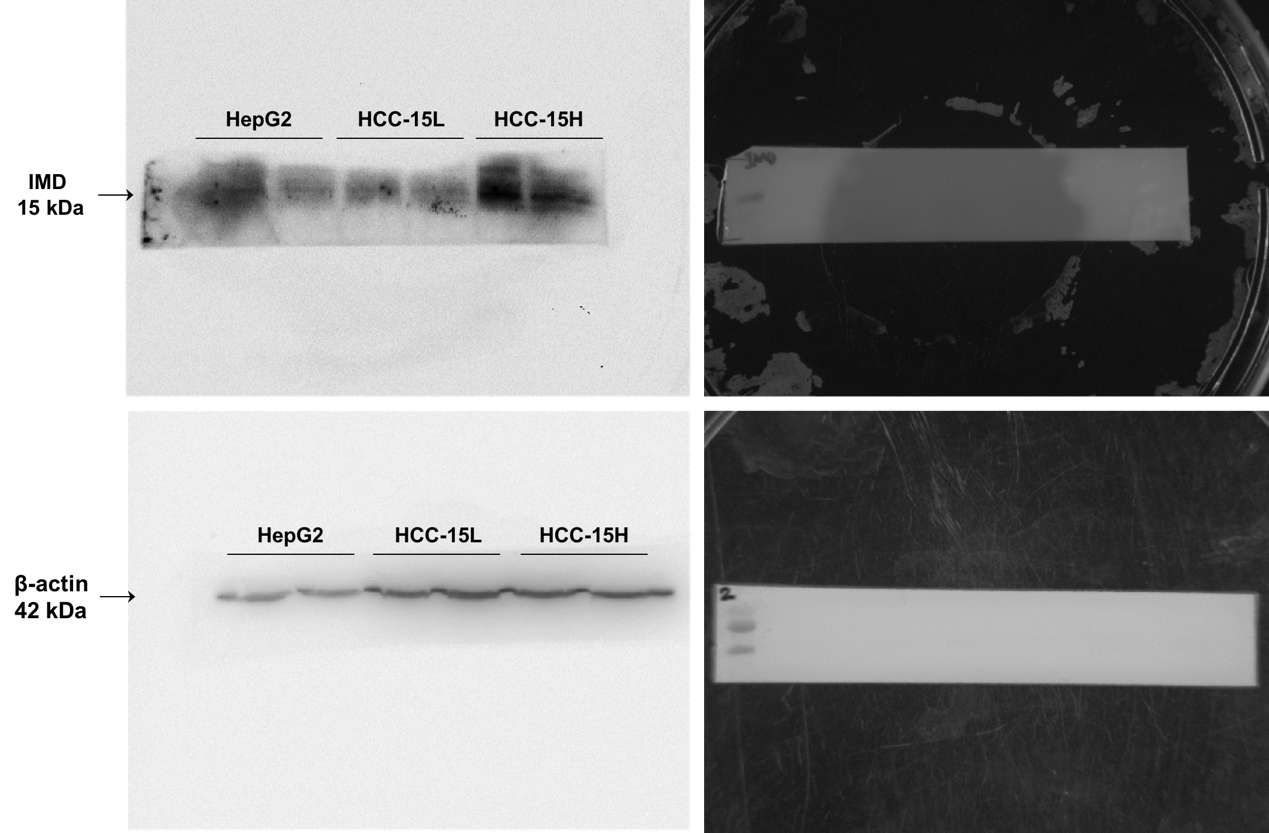


**Supplementary Fig S6. The original uncropped WB bands of Fig 2f.** The expression of IMD in HepG2 cells was cropped from the gel. In Fig 2f, only the blots of IMD expression in HCC-15L and HCC-15H were shown.


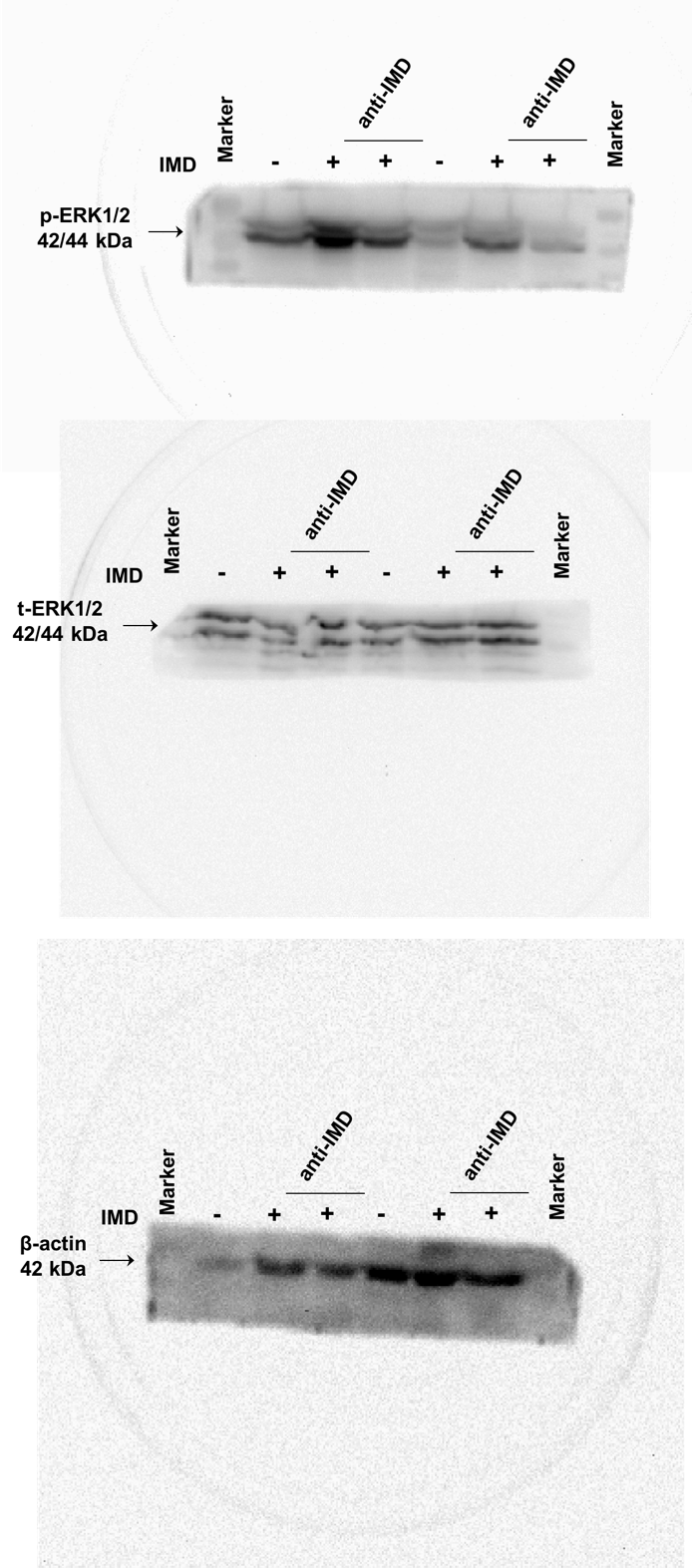


**Supplementary Fig S7. The original uncropped WB blots of Fig 6i.**
